# Intraoperative and Postoperative Outcomes in Patients Undergoing Total Laparoscopic Hysterectomy for Benign Conditions With Drain Placement

**DOI:** 10.7759/cureus.80978

**Published:** 2025-03-22

**Authors:** Nur Derya Malkan, Hilal Aktürk, Cansu Tekin, Sadık Şahin

**Affiliations:** 1 Obstetrics and Gynecology, Adiyaman Kahta State Hospital, Istanbul, TUR; 2 Obstetrics and Gynecology, Muş Malazgirt State Hospital, Istanbul, TUR; 3 Obstetrics and Gynecology, Zeynep Kamil Women's and Children's Diseases Training and Research Hospital, Istanbul, TUR

**Keywords:** drain insertion, laparoscopic gynecological surgery, laparoscopy, total laparoscopic hysterectomy (tlh), transfusion

## Abstract

Introduction: Hysterectomy is a common gynecological procedure, with laparoscopic techniques increasingly favored for their minimally invasive nature. The use of drains during total laparoscopic hysterectomy (TLH) remains controversial, with conflicting evidence regarding their efficacy and impact on outcomes.

Materials and methods: This retrospective study analyzed data from 415 patients who underwent TLH for benign indications at Istanbul Zeynep Kamil Women's and Children's Diseases Training and Research Hospital between 2020 and 2022. Patients were categorized into two groups based on drain application. Demographic and clinical data were collected, and perioperative and postoperative outcomes were compared between groups.

Results: Of the 415 patients, 277 (66.7%) received drains during TLH. Analysis revealed that patients who received drains had significantly longer operation times, an increased length of hospital stay, higher complication rates, and a greater need for transfusion than those without drains. Although preoperative hemoglobin levels were similar between groups, postoperative levels were significantly lower in drain recipients. However, there was no significant difference in postoperative gas discharge rates between groups.

Conclusions: This study suggests drain application during TLH may predict bleeding complications and influence perioperative outcomes. Despite limitations, including their retrospective nature, the findings contribute to the ongoing debate surrounding drain use in TLH and provide valuable insights for clinical practice. Further prospective studies are warranted to validate these findings and guide evidence-based decision-making in gynecological surgery.

## Introduction

Hysterectomy is the second most frequently performed major surgical procedure in gynecology, following cesarean section [1]. Approximately 70% of hysterectomies worldwide are performed for non-cancerous reasons, including irregular uterine bleeding, uterine fibroids, and uterine prolapse [2]. The mean age of women undergoing hysterectomy is 46.1 years, with the majority falling within the age range of 20 to 49 years [3]. A multicentric retrospective analysis showed an initial increase in hysterectomy rates until 1985, followed by a subsequent decline. Concurrently, there was a reduction in the length of hospital stays [4].

For the past century, abdominal laparotomy incisions have been the most common method for performing hysterectomies. However, data indicate that vaginal hysterectomy is the preferred method for the removal of benign uterine disease.

A 2015 Cochrane review analyzing 47 studies involving 5,102 patients who underwent different types of hysterectomy (vaginal, abdominal, and laparoscopic) found that vaginal hysterectomy resulted in quicker recovery and an improved quality of life compared to abdominal hysterectomy [5]. Vaginal hysterectomy also demonstrated greater efficiency compared to laparoscopic hysterectomy, as evidenced by shorter surgery durations and hospitalization periods, leading to enhanced cost-effectiveness. Numerous international professional organizations advocate for vaginal hysterectomy as the preferred surgical method, reserving laparoscopic hysterectomy for cases where a vaginal approach is not feasible.

The use of drains in surgical procedures has been a subject of ongoing debate for an extended period [6]. In recent years, there has been a widespread tendency to avoid using drains in surgeries [7]. The primary purpose of applying a leading drain is to anticipate postoperative complications and prevent the recurrence of abscess formation [8]. Several surgical clinics have researched the necessity of postoperative drainage to remove residual tissues and fluids [9]. Some researchers suggest that drainage can alleviate surgical pain by releasing trapped gas in the abdomen, while others emphasize that its use may not significantly reduce postoperative pain [10].

Despite the benefits of drainage, there are also adverse effects. One example is the formation of a hernia at the drainage site, which may require surgical intervention [11]. Additionally, drains are associated with prolonged hospital stays and increased surgery durations [12].

This study aimed to investigate the use of drains in total laparoscopic hysterectomy (TLH) procedures performed at our institution, focusing on their impact on perioperative and postoperative outcomes.

## Materials and methods

This study included patients who underwent TLH for non-cancerous reasons at the Istanbul Zeynep Kamil Women's and Children's Diseases Training and Research Hospital between 2020 and 2022. The patients were categorized into two groups based on whether drains were used during the surgical procedure. The study group consisted of patients who underwent TLH with drain placement, while the control group comprised patients who underwent the same procedure without drains. The study aims to compare and analyze the perioperative and postoperative outcomes associated with drain use in TLH cases by categorizing patients into these two groups.

The eligibility criteria for this study included women aged 35 to 65 who underwent TLH for specific gynecological conditions such as myoma uteri, abnormal uterine hemorrhage, or endometriosis. Additionally, patients who underwent TLH with either bilateral or unilateral salpingo-oophorectomy were considered. By specifically targeting these demographic and surgical characteristics, this study focuses on a homogeneous population representative of individuals who commonly undergo TLH for benign conditions.

Exclusion criteria were established to ensure the relevance and specificity of the research. Female patients younger than 35 or older than 65 were excluded. Additionally, women who underwent TLH for malignant or urogynecological conditions were not included. Patients who underwent additional procedures during the same surgical session were also excluded. The indications for TLH were carefully assessed through a detailed medical history review, vaginal examination, and imaging techniques such as transabdominal and transvaginal ultrasound at the hospital's gynecological outpatient clinic. After completing preoperative evaluations, eligible patients underwent TLH in the hospital's operating room. The strict exclusion criteria were implemented to ensure the validity and reliability of the study findings within the defined scope.

Prior to commencing the retrospective assessment of patient records, the Ethics Committee of Zeynep Kamil Women's and Children's Diseases Training and Research Hospital approved the study (approval number: 02-01/2022). Patient data were obtained from the hospital information system, as well as from patient and anesthetic records. The collected data included the following variables: age, gravidity, parity, mode of delivery, history of previous abdominal surgery, presence of systemic diseases, drain application, amount of fluid drained (in milliliters), day of drain removal, days until gas-stool discharge, indication for hysterectomy, performance of oophorectomy (unilateral or bilateral), pathology results, operation duration, preoperative and postoperative day 1 hemoglobin levels, length of hospital stay, vascular, intestinal, and bladder injuries, vaginal cuff hematoma, postoperative vaginal bleeding, wound site infection, and whether blood or blood product transfusion was performed. The operation duration was between making the incision and closing the wound. The duration of hospitalization, the time until drain removal, and the time until gas-stool release were assessed in days.

Our facility maintains an intra-abdominal CO₂ pressure of 12 mmHg during laparoscopic hysterectomies. Additionally, drains are implanted through a 5 mm port in the left or right inguinal area. The drainage system is a 14-ch nasogastric tube with a single aperture at the distal end, introduced into the Douglas pouch using a laparoscopic atraumatic device. Prior to surgery, patients scheduled for a TLH are required to be admitted to the hospital one day in advance. Prophylactic anticoagulation is administered when necessary. Each patient receives 1 g of cefazolin as preoperative prophylaxis, and postoperatively, standard instructions are provided regarding antiemetics and analgesics. Patients are categorized into groups based on whether drains were used, and all collected data are statistically analyzed to compare intraoperative and postoperative outcomes between these groups.

This study complies with the Helsinki Declaration and principles of good clinical practice and does not violate ethical standards for research involving human subjects. The confidentiality of patient identities and medical records will be upheld, and no external parties will have access to this information except for the researchers. The researchers do not receive any secondary benefits from conducting this study.

Statistical analysis

Continuous variables are typically presented as the mean ± standard deviation or, alternatively, as the median, minimum, and maximum range. Categorical data, on the other hand, is reported as the number of occurrences and the corresponding percentage. The normality of continuous variables was assessed using the Kolmogorov-Smirnov goodness-of-fit test. The Student's t-test was used to analyze between-group differences for variables that followed a normal distribution. At the same time, the Mann-Whitney U test was applied to variables that did not follow a normal distribution. Categorical data comparisons were performed using the Chi-square or Fisher's exact tests. Statistical analysis used SPSS Statistics version 26.0 (IBM Corp. Released 2019. IBM SPSS Statistics for Windows, Version 26.0. Armonk, NY: IBM Corp). A p-value of less than 0.05 was considered statistically significant.

## Results

In Table 1, analyses of 415 patients revealed a median age of 49 years (range: 35-65) and a parity value of 2 (range: 0-8). Among them, the number of cases with a not specified date (NSD) was 138 (33.3%), with 128 (30.8%) having a history of previous surgeries, 300 (72.3%) undergoing oophorectomy, and 277 (66.7%) receiving drain application. A total of 214 (77.3%) drains were removed within the first 24 hours, 56 (20.2%) within 48 hours, and seven (2.5%) after 48 hours. Zero indicates no oophorectomy, 1 indicates bilateral oophorectomy, and 2 indicates unilateral oophorectomy (Table 2).

**Table 1:** Clinical characteristics of patients

Demographic and clinical characteristics	Median (min-max)
Age (year)	49 (35-65)
Parity	2 (0-8)

**Table 2:** Demographic data related to the patients

Demographic data		N	%
Vaginal birth	0	80	19
	1	45	10
	2	138	33.3
	3	86	20.7
	4	33	8.0
	5	17	4.1
	6	9	2.2
	7	5	1.2
	8	2	0.5
Previous operation	None	287	69.2
	Yes	128	30.8
Drain application	None	138	33.3
	Yes	277	66.7
Drain removed	Day 1	214	77.3
	Day 2	56	20.2
	Day 3	7	2.5
Oophorectomy	0	101	24.3
	1	300	72.3
	2	14	3.4
Total		415	100.0

In our current study, indications related to the patient are mentioned in Table 3: resistant abnormal uterine bleeding was observed in 166 (40.0%) cases, abnormal uterine bleeding coexisting with uterine myoma in 41 (9.9%) cases, and abnormal uterine bleeding associated with tamoxifen use in 19 (4.6%) cases. Consequently, a decision for surgery was made.

**Table 3:** Indications related to the patients AUB: abnormal uterine bleeding, PMB: post-menopausal bleeding

Operation indications	N	%
Treatment-resistant AUB	166	40.0
AUB + myoma uteri	41	9.9
AUB + tamoxifen use	19	4.6
PMB	59	14.2
Myoma uteri	37	8.9
Adnexal mass	53	12.8
Endometrial hyperplasia	15	3.6
Cervical dysplasia	19	4.6
Other	6	1.4

The examination of the surgical material extracted postoperatively revealed the following pathological findings: adenomyosis was detected in 65 (15.7%) cases, atypical endometrial hyperplasia in six (1.4%) cases, atrophic endometrium in one (0.2%) case, simple ovarian cysts in 23 (5.6%) cases, simple ovarian cysts and myoma in 16 (3.9%) cases, and simple ovarian cysts and atrophic endometrium in one (0.2%) case (Table 4).

**Table 4:** Pathology results for patients EIN: endometrial intraepithelial neoplasia, PC: Pipelle curettage, HSIL: high-grade intraepithelial lesion, LSIL: low-grade squamous intraepithelial lesion

Pathology results	N	%
Adenomyosis	65	15.7
Endometrial hyperplasia without atypia	6	1.4
Atrophic endometrium	1	0.2
Simple ovarian cysts	23	5.6
Simple ovarian cysts + myoma	16	3.9
Simple ovarian cysts + atrophic endometrium	1	0.2
Simple ovarian cysts + adenomyosis	2	0.5
Benign pathology	53	2.8
EIN (Pc normal)	2	0.2
Endometrial polyp	42	10.1
Endometrial polyp + myoma	1	0.2
Endometrioma	5	1.2
Endometriosis + myoma	5	1.2
Endometriotic cyst + endometrial polyp	1	0.2
Endometrium Ca (PC normal)	3	0.7
Focal EIN + myoma	1	0.2
Endometrium under the influence of gestagen	1	0.2
HSIL	7	1.7
LSIL	2	0.5
LSIL + myoma	2	0.5
Simple myoma uteri	169	40.6
Myoma + EIN	1	0.2
Myoma + endometrial polyp	1	0.2
Paratubal cyst	1	0.2
No residual lesion	1	0.2

Statistical analysis revealed significant differences in operation duration, hospital stay, complication rates, and transfusion rates between patients with and without drains (p=0.003, p=0.010, p=0.032, and p=0.033, respectively). Conversely, additional diseases, oophorectomy, and postoperative gas discharge rates did not differ significantly between the groups (p>0.05). Zero indicates no oophorectomy, 1 indicates bilateral oophorectomy, and 2 indicates unilateral oophorectomy (Table 5).

**Table 5:** Comparison of some clinical characteristics between patients undergoing and not undergoing drainage * Mann-Whitney U test, ** Student’s t-test, *** Fisher's exact test, **** Chi-square test SD: standard deviation

Clinical characteristics		Drain (-) (n=138)	Drain (+) (n=277)	p-value
Operation time (minutes) (median (min-max))		90 (40-180)	100 (40-300)	0.003*
Duration of hospital stay (days) (mean ± SD)		2.93 ± 0.91	3.20 ± 1.02	0.010**
Complications (n, %)	None	129 (93.5%)	239 (86.3%)	0.032***
	Yes	9 (6.5%)	38 (13.7%)	
Transfusion (n, %)	None	138 (100.0%)	268 (96.8%)	0.033***
	Yes	0 (0.0%)	9 (3.2%)	
Comorbidities (n, %)	None	114 (82.6%)	227 (81.9%)	0.869****
	Yes	24 (17.4%)	50 (18.1%)	
Oophorectomy	0	30 (21.7%)	71 (25.6%)	0.393****
	1	105 (76.1%)	195 (70.4%)	
	2	3 (2.2%)	11 (4.0%)	
Postop with gas discharge (days)	1	135 (97.8%)	266 (96.0%)	0.403***

Among the 415 patients, three (0.72%) were diagnosed with cuff infections, with two (0.48%) managed as outpatients using oral antibiotics. Within 16 weeks postoperatively, six patients (1.45%) presented with vaginal bleeding, and cuff hematoma was identified in all six cases. One patient (0.24%) required re-suturing due to bleeding at the cuff site. Two patients (0.48%) developed trocar site infections managed with outpatient antibiotic therapy. One patient (0.24%) experienced a cerebrovascular event, and another (0.24%) suffered a pulmonary embolism; both required admission to the intensive care unit. Five patients (1.20%) were diagnosed with bladder injuries, which were repaired intraoperatively with urology consultations. One patient (0.24%) developed postoperative ileus, which was managed medically. Another patient (0.24%) experienced a bowel serosal injury, necessitating a general surgery consultation for serosal repair.

Preoperative hemoglobin levels were slightly lower in patients with drains than those without, but the difference was insignificant (p=0.059). However, postoperative hemoglobin levels were significantly lower in patients with drains (p=0.036). In both groups (with and without drains), postoperative hemoglobin levels were significantly lower than preoperative levels (p<0.001 for both) (Table 6).

**Table 6:** Comparison of hemoglobin levels in drainage and non-drainage patients * Mann-Whitney U test, ** Wilcoxon sequential signs test

Surgical procedure status	Drain (-) (median (min-max)) (n=138)	Drain (+) (median (min-max)) (n=277)	p-value
Preop. Hb. (g/dL)	12.5 (7.8-15.9)	12.2 (8.4-16.3)	0.059*
Postop. Hb. (g/dL)	10.8 (6.9-14.7)	10.4 (1.1-14.1)	0.036*
	p<0.001**	p<0.001**	

## Discussion

Hysterectomy is a commonly performed surgical procedure in gynecology. Currently, surgical techniques are advancing toward minimally invasive methods. Laparoscopic hysterectomy is superior to abdominal hysterectomy due to several advantages, including reduced hospitalization time, faster recovery, improved aesthetic outcomes, decreased postoperative pain, and a lower incidence of wound infection. As a result, laparoscopic hysterectomy is the preferred option. The choice of hysterectomy technique depends on various factors, such as indications, accompanying pathology, other medical conditions, and the surgeon's level of expertise. Hysterectomy is often selected as a preferred treatment for multiple conditions, including uterine leiomyomas, abnormal uterine bleeding, malignant and premalignant diseases, infection-related chronic pelvic pain, and prolapse.

The use of drains in gynecological procedures has been debated for many years. According to current Enhanced Recovery After Surgery (ERAS) guidelines, drains should not be used in gynecological surgeries unless absolutely necessary. Additionally, if drains are used, they should be removed as soon as possible. However, there remains a lack of consensus in clinical practice and academic literature. While numerous studies have examined the impact of drain usage on postoperative pain in laparoscopic hysterectomy, definitive evidence regarding factors such as hemoglobin fluctuations, transfusion requirements, and complications is still lacking.

Our study found that 66.7% of the participants had drains applied. We categorized the patients into two groups based on whether or not they received drainage and then evaluated intraoperative and postoperative outcomes accordingly. Analysis of the demographic data revealed that the mean patient age was 49 years, with an average gravidity of three and an average of two deliveries. Drainage was performed in 277 out of 415 patients. The mean evacuated fluid volume was 80 cc, with 214 drains removed within the first 24 hours, 56 drains removed within 48 hours, and seven drains removed after more than 48 hours.

A study conducted in 2021 included 445 individuals. The study aimed to analyze the retrospective indications for surgery and the clinical outcomes of these patients. The primary indications for hysterectomy were irregular uterine hemorrhage and uterine fibroids [13]. Our study analyzed 639 individuals who underwent laparoscopic hysterectomy at our hospital over the past three years. A total of 224 cases were excluded from the study due to their malignant nature or because they underwent an additional procedure during the same session. In line with the research conducted in 2021, our study likewise identified abnormal uterine hemorrhage and uterine fibroids as the primary reasons for performing hysterectomy.

A comprehensive evaluation conducted in 2014 analyzed 216 studies to assess the outcomes of peritoneal gas drainage in gynecological procedures. The study focused on managing shoulder discomfort, the need for analgesics, and the overall postoperative pain experience. While there were no significant differences in the requirement for pain relief medication and anti-nausea drugs between the control and experimental groups, individuals who received drainage experienced a substantial reduction in shoulder discomfort and total postoperative pain. Our study found that among the 277 patients who had drainage, 266 experienced gas release within the first 24 hours, while 11 patients experienced it within the first 48 hours. Among the 138 patients who did not receive drainage, 135 experienced gas release within the first 24 hours, while three experienced it within the first 48 hours. There was no significant difference between the two groups.

In a randomized, controlled prospective study conducted in 2002, the investigation found no significant differences between the drain and non-drain groups in terms of operation time, estimated blood loss, postoperative hemoglobin levels, complication rates, or hospital stay [14]. However, our investigation revealed decreased hemoglobin levels after surgery in individuals who underwent drainage, in contrast to those who did not. Additionally, patients who received drainage experienced higher complication rates and required more transfusions.

Our study revealed that preoperative hemoglobin levels were lower in patients who received drainage than in those who did not. However, it is important to note that this difference did not reach statistical significance. Furthermore, patients who underwent drainage experienced a substantial decrease in postoperative hemoglobin levels compared to those who did not. Both groups, those who received drainage and those who did not, experienced a statistically significant decrease in postoperative hemoglobin levels compared to their preoperative levels (p<0.001 for both groups).

According to our research, surgeons tend to use drains in patients with low hemoglobin levels before surgery, which may increase the risk of bleeding during the operation. Our findings differ from those of Shen et al., as we observed statistically significant increases in operation duration, length of hospital stay, complication rates, and transfusion rates among patients who received drainage compared to those who did not (p=0.003, p=0.010, p=0.032, and p=0.033, respectively) [14].

A study conducted in 2016 examined the use of drainage in gynecological procedures performed for non-malignant conditions. Patients were categorized into two groups based on whether the operation duration exceeded or was less than two hours. Within the group with an operation time of less than two hours, patients were further divided into those who received drainage and those who did not, resulting in experimental and control groups [15]. Statistically significant differences were observed in postoperative pain levels between the drainage and control groups, with the most significant difference occurring at the sixth hour after surgery. However, no significant differences were observed in postoperative complication rates or length of hospital stay.

A study conducted in 2018 included 2,013 patients who underwent laparoscopic hysterectomy. The study aimed to compare the occurrence of complications during surgery. A total of 184 patients experienced complications, including two with ureteral injuries, 11 with bladder injuries, nine with vascular injuries, eight with intestinal injuries, and four who required blood transfusions [16].

Limitations of the study

This retrospective study at Istanbul Zeynep Kamil Women's and Children's Diseases Training and Research Hospital provides insights into perioperative and postoperative outcomes associated with drain usage in TLH cases. However, it has inherent limitations as a single-institution study with a retrospective design and a limited sample size. While the exclusion criteria were essential for maintaining homogeneity, they may have introduced selection bias, potentially overlooking important patient subgroups. Variability in drain utilization among surgeons and the lack of consideration for certain confounding variables could affect the study's internal validity. Furthermore, the focus on perioperative and postoperative outcomes may overlook long-term complications and patient-reported outcomes. Although appropriate statistical methods were employed, the chosen significance threshold and the potential for type I or II errors should be considered. Ongoing ethical oversight remains crucial, despite claims of compliance with ethical standards. Finally, publication bias may influence the overall interpretation of the literature on drain utilization in laparoscopic hysterectomy.

## Conclusions

The debate surrounding the use of drains in total laparoscopic hysterectomies has persisted over time. Our study explored this contentious issue by examining intraoperative and postoperative outcomes associated with drain application. Notably, our analysis revealed a significant disparity in complication rates, length of hospital stay, and transfusion requirements between patients who underwent drain placement and those who did not. Furthermore, while preoperative hemoglobin levels showed no statistically significant difference between the two groups, postoperative hemoglobin levels were markedly lower in patients who received drains, indicating a potential association between drain use and postoperative bleeding.

Overall, our findings underscore the role of drain application as a predictive tool for bleeding complications following total laparoscopic hysterectomies, highlighting its clinical significance in optimizing patient outcomes.
